# Optimization and comparative study of *Bacillus* consortia for cellulolytic potential and cellulase enzyme activity

**DOI:** 10.1515/biol-2025-1066

**Published:** 2025-03-06

**Authors:** Ogechukwu Bose Chukwuma, Mohd Rafatullah, Riti Thapar Kapoor, Husnul Azan Tajarudin, Norli Ismail, Mahboob Alam, Masoom Raza Siddiqui

**Affiliations:** Environmental Technology Division, School of Industrial Technology, Universiti Sains Malaysia, Penang, 11800, Malaysia; Centre for Plant and Environmental Biotechnology, Amity Institute of Biotechnology, Amity University Uttar Pradesh, Noida, 201 313, Uttar Pradesh, India; Bioprocess Technology Division, School of Industrial Technology, Universiti Sains Malaysia, Penang, 11800, Malaysia; Division of Chemistry and Biotechnology, Dongguk University, 123, Dongdaero, Gyeongju-si, 780714, Republic of Korea; Chemistry Department, College of Science, King Saud University, Riyadh, 11451, Saudi Arabia

**Keywords:** *Bacillus* species, cellulolytic ability, consortia, landfill leachate, lignocellulose biomass

## Abstract

Lignocellulosic biomass, owing to its recalcitrant nature, requires a consortium of enzymes for its breakdown. The present study deals with the isolation of cellulolytic bacterial strains from landfill leachate collected from the Pulau Burung landfill site of Penang, Malaysia, and consortia were constructed to test their cellulolytic efficiency. The dinitro salicylate method was used for the estimation of enzyme activity, and consortia were compared with promising bacterial strains. The combined potential of promising bacterial strains was optimized at varying experimental conditions to detect their maximum cellulolytic activity. The results showed that eight bacterial strains reflected hydrolytic activities, and these were identified by 16S rDNA sequence as *Bacillus subtilis, Bacillus pumilus, Bacillus proteolyticus, Bacillus paramycoides, Bacillus cereus, Bacillus altitudinis, Bacillus niacin,* and *Bacillus thuringiensis.* Consortia A included *Bacillus proteolyticus, Bacillus subtilis, Bacillus pumilus,* and *Bacillus paramycoides* and reflected high thermophilic inclination as the optimal temperature was 45°C at pH 6 with the highest cellulase activity of 0.90 U/ml. Consortia B included *Bacillus cereus, Bacillus altitudinis, Bacillus niacin,* and *Bacillus thuringiensis* and showed a cellulase activity of 0.78 U/ml at 38°C and pH 6. The results reflected the significant potential of these *Bacillus* strains and consortia in the breakdown of cellulose into useful end products. The consortia further proved that a synergistic relationship was more favourable for bioconversion processes.

## 1 Introduction

The high energy demand, exhaustion of fossil fuel reserves, industrialization, and a significant increase in agricultural or municipal solid waste have turned global efforts towards the exploration of renewable sources for use in the generation of green energy and better waste management strategies [1]. Approximately 200 million tonnes of lignocellulosic wastes are generated every year through various agricultural activities [2]. Lignocellulosic wastes are cheap and widely available at zero cost and have a great potential for biotransformation into biofuels such as bioethanol, biogas, and other value-adding products [3,4,5]. The lignocellulosic wastes are composed of cellulose (30–50%), hemicellulose (15–30%), and lignin (10–20%) [6]. Various methods like physical, biological, and chemical procedures have been used for the conversion of lignocellulosic wastes [7]. The application of gas or steam utilizes high pressure alongside high temperature to make lignocellulose softer and break the chemical linkages between the cellulose components [8]. During this process, many harmful intermediates like furfural or its derivatives and acetic acid are also produced. The chemical method, such as acid or base treatment, breaks down glycosidic bonds, shows a reduction in cellulose polymerization, and enhances its breakdown. However, physical and chemical methods are effective, but these procedures are unsustainable due to the adverse effects on the environment using toxic chemicals and high energy consumption [9,10].

The biological method works with microbes having efficient enzymes, which can easily degrade cellulose and hemicellulose. It utilizes microbial enzymes to break down the lignocellulosic polysaccharides by enzymatic hydrolysis and fermentation [11]. As compared to other procedures, the application of microbes is safe, cost-effective, feasible, environmentally benign, consumes less energy, requires mild conditions, does not produce detrimental by-products, and can be applied to several industrial applications [12,13,14]. Microbial cellulases are important biocatalysts and can be used for the degeneration of lignocellulosic wastes, biofuel generation, and biorefining [5,15]. Cellulase is the main enzyme used in the depolymerization of lignocellulosic polysaccharides, which involves the breakdown of β-1,4 bonds of cellulose to generate glucose molecules [16]. Cellulase is made up of exoglucanases, endoglucanases, and *β*-glucosidases, which operate in synergy with one another in the hydrolysis of lignocellulosic biomass [17]. Cellulase is the most in-demand enzyme, which covers about 20% of the global market [13]. Microbial enzymes have been proven very efficient for the bioconversion of lignocellulosic waste compared to commercial enzymes [18]. They are a cost-effective option as opposed to available commercial and pure enzymes [19]. Transformation of lignocellulosic wastes into biofuel via cellulases can combat the problems of consumption of fossil fuel and release of greenhouse gases, which are main global concerns [20,21].

Bacteria are easily and quickly culturable, have less nutritional requirements, and produce large quantities of cellulase enzymes [22]. Bacteria are prolific producers of cellulase and are considered to be suitable candidates for cellulose breakdown due to their fast reproducibility and ability to recombine [12]. The generation of cellulase has been previously reported by different genera of anaerobic, mesophilic, aerobic, and thermophilic bacteria such as *Bacillus, Cellulomonas, Thermomonospora, Acetovibrio, Microbispora, Fibrobacter, Streptomyces, Ruminococcus, Paenibacillus,* etc. [23,24]. *Arthrobacter woluwensis* TDS9 played a significant role in cellulolytic biomass saccharification [4]. *Bacillus* genus is a promising enzyme-producing candidate with high catalytic potential and cellulose degradation efficiency even under harsh environmental conditions [1,25,26].

The bacterial consortia have been proven functionally robust, efficient in enzyme production, and stable against biochemical degradation compared to the single bacteria [27]. Constructed consortia are co-cultures that contain various bacterial species or different strains from the same species. The cellulolytic enzymes produced by different bacteria present in consortia act in synergy, and this leads to the complete breakdown of lignocellulosic wastes [28]. Zerva et al. [29] stated that the complete breakdown of various components of lignocellulosic wastes requires a consortium of enzymes that perform different roles in the degradation process. There are reports that tasks that single culture might find tedious due to more consumption of time were easily achieved by bacterial consortia for lignin and cellulose degradation and generation of valuable bio-products [30]. Furthermore, inhibition by intermediates and thermodynamic challenges can be overcome by using the principles of division of labour. Co-culturing can help in reducing the steps involved in pre-treatment and enzyme production, which can be integrated into one step to reduce expenditure in the process. Vu et al. [30] found that consortia that were formed by the combination of two to three *Bacillus* species showed enhanced enzymatic hydrolysis efficiency on wheat bran compared to the monoculture of each strain. *Bacillus* strains like *B. velezensis, B. toyonensis,* and *B. safensis* reflected high cellulolytic activities [31]. *Bacillus coagulans, B. cereus, B. subtilis*, and *B. licheniformis* have been identified with high cellulolytic efficiency [30]. Various *Bacillus* species have been observed to produce cellulase enzymes that breakdown recalcitrant lignocellulosic biomass; however, fewer studies have been conducted on mixed microbial culture for their degradation efficiency.

Landfill sites contain a huge amount of lignocellulosic material, heterogeneous in nature and are reported as a suitable site for biomass transformation due to the presence of potential indigenous microbes. Unfortunately, landfill sites have not been extensively studied yet [32]. Previous investigations have confirmed that *Bacillus* species secrete enzymes for lignin and cellulose degradation, break the biphenyl structure of lignin, and metabolize dioxane lignin. To the best of our knowledge, no reports are available on the isolation of potential bacteria and formulation of bacterial consortia for the conversion of lignocellulose wastes from the Pulau Burung landfill site in Malaysia. Thus, in this study, the cellulolytic ability of *Bacillus* strains isolated from the Pulau Burung landfill site and two consortia formed by using knowledge from the previous metagenomic study of the same landfill site was applied and analysed [33]. The present investigation is unique as it combines potential bacterial strains and optimization of different experimental parameters for maximum cellulolytic activity. The objective of the present investigation was (i) to use information from a previous study as a guide in the design of bacterial consortia, (ii) compare cellulolytic responses of a single *Bacillus* strain against constructed consortia, and (iii) generate information and recommendations for the use of potential bacterial strains and consortia in lignocellulose-driven enterprise in the future.

## 2 Materials and methods

### 2.1 Sampling site

The samples of landfill leachate were procured from the Pulau Burung landfill area (5°19.36;100°42.67′E) of Penang, Malaysia, during the summer season in July. As per the standard procedures, samples were transferred to the laboratory, and physicochemical and microbiological analyses were carried out using the Forster method [34].

### 2.2 Isolation and identification of bacteria

Bacterial species were isolated by serially diluting 1 ml of leachate tenfold, and then dilutions (0.1 ml) from each fold were spread on sterile nutrient agar plates and kept at 37°C for a day. To ensure purity prior to identification, the developed colonies were sub-cultured. Pure bacterial colonies were placed at 4°C on nutrient agar slants for investigation. The morphology of the colonies was observed, and the similar colonies were sub-cultured until pure colonies were obtained. After the isolation of bacteria, morphological and biochemical characterization were performed by following standard methods prescribed in Bergey’s Manual of Determinative Bacteriology for screening of promising bacteria [35]. Morphological characterization was conducted based on the visual appearance and Gram staining.

Gram staining was done by using bacteria that were grown overnight, and microscopic characterization was used to affirm colony characteristics. According to the procedure of Smith and Hussey [36], a glass slide was smeared with the culture by placing a few drops of distilled water on the slide, and a thin layer of bacterial colony was spread in the drop before fixing with heat. This was followed by treatment with crystal violet and then rinsing with distilled water. Then, the slides were flooded with Gram’s iodine solution before being rinsed with alcohol. Finally, the slide was flooded with safranin, washed with distilled water, left to air dry, and then observed under a microscope with a magnification of 100× objective lens using immersion oil.

### 2.3 Qualitative and quantitative screening for cellulolytic abilities

The extracellular cellulase activity was measured using the procedure of Hajiabadi et al. (2020). Carboxymethyl cellulose (CMC) agar medium (g/L) (CMC, 5.0; NaNO_3_, 2.5; yeast extract, 1.0; KH_2_PO_4_, 1.0; NaCl, 1.0; MgSO_4_·7H_2_0, 0.6; CaCl_2_, 0.1; FeCl_3_, 0.1; agar, 15.0) was used and pH 7 was maintained by adding 1 M NaOH solution. The plates were inoculated with the pure strains, incubated for 48 h, and then treated with Congo red and Gram’s iodine staining procedures. The colonies on the CMC agar were treated with the respective dyes. Gram’s iodine reagent was washed with water, while Congo red was rinsed with 1 M NaCl solution. Based on the cellulolytic activity, organisms were selected for further screening. The test was repeated for strains that showed clearance zones in both Congo red and Gram’s iodine. In this quantitative part, the zones of clearance were measured to determine the strains that showed higher activity and those that showed the lowest activity.

### 2.4 Effect of temperature on cellulase activity

To carry out a preliminary check on the impact of temperature on the growth of bacteria and cellulolytic activity, isolates were grown on agar plates at different temperatures (28, 37, and 45°C). After this, Gram’s iodine reagent was used for staining plates and to observe the zone of clearance around the bacterial colony.

### 2.5 Growth curve

For assessment of the growth pattern of bacterial strains, they were inoculated in nutrient broth and studied at varying temperatures. The flasks were agitated at 150 rpm, and the absorbance was measured at 620 nm after 24 h by using a UV-Vis spectrophotometer.

### 2.6 Molecular identification of the selected bacterial strain

After the study on cellulase activity, the best isolates were selected for molecular biology techniques. Bacterial isolates were freshly grown in the nutrient broth and kept overnight at 37°C, under shaking conditions at 150 rpm. DNA was extracted from pure bacterial isolates as per the procedure of Yi et al. [37] by using a Vivantis DNA kit (Vivantis, Selangor, Malaysia). The extracted DNA underwent amplification by the polymerase chain reaction by employing universal DNA forward Eubac27F and reverse 1492R primers to earmark bacterial 16S rRNA. Then, the PCR protocol was applied: 32 cycles at 95°C for 3 min, 94 and 56°C for 1 min, 72°C for 2 min, and 72°C for 10 min, with an interval of 4°C. The libraries were normalized and pooled according to the protocol recommended by Illumina, followed by sequencing using the MiSeq platform with 300 PE. The sequences obtained were analysed by applying NCBI’s online tool, Blast.

### 2.7 Phylogenetic analysis

Blasting was performed by the use of the GenBank database for the collation of 16S rDNA sequences with similar types of sequences. Molecular Evolutionary Genetics Analysis (MEGA) software was used to construct the phylogeny of bacterial strains with the help of the obtained sequences.

### 2.8 Development of bacterial consortium

Bacterial consortia were prepared by combining equal proportions of cell cultures of the best bacterial isolates. The culture of *B. proteolyticus*, *B. subtilis*, *B. pumilus*, and *B. paramycoides* kept for 24 h was mixed to form consortia A, whereas the culture of *B. cereus*, *B. altitudinis*, *B. niacini*, and *B. thuringiensis* was mixed to form consortia B. The objective behind choosing and mixing specific bacterial strains was to form consortia A and B by selecting promising indigenous strains with better cellulolytic activities present at landfill sites. The interaction of promising bacterial species in consortia may provide desired results compared to the individual isolates.

### 2.9 Quantitative screening and optimization for cellulolytic abilities

Cellulase activity assay was carried out as per the procedure of Ghose [38] by measuring the released reducing sugar with a dinitrosalicylic acid reagent. The absorbance was measured with a UV-vis spectrophotometer at 540 nm. The reduced sugar content was determined by using a standard curve for glucose. The process optimization was conducted by fermentation of bacterial strains and consortia at (i) pH 6 and 8 and (ii) temperatures of 32, 38, 45, and 50°C, and the cellulase activity was measured and analysed.

### 2.10 Optimization of the biodegradation process using RSM

The random surface methodology was applied for optimization of the most promising bacterial consortia. The main objective of the RSM application was the optimization of output variables that depend on input variables by the experimental design. Cellulase activity response was directly dependent on three input variables: temperature, pH, and substrate concentration, which were also analysed.

### 2.11 Statistical analysis

All the experiments were conducted in triplicate, and data are presented as mean ± standard error. A two-factor ANOVA test with significance level *P* < 0.05 was used for data analysis. Design expert software and an origin plot were used to generate the figures.

## 3 Results and discussion

### 3.1 Identification and isolation of bacteria

A total of 19 isolates were reported, which were microscopically identified for morphological characterization. The Gram-negative bacteria were decolourized after alcohol treatment and appeared red, whereas Gram-positive bacteria did not decolourize and reflected purple colour under a microscope. Gram staining results showed that 12 strains were Gram-positive, whereas 7 were Gram-negative strains, similar to the results of our previous investigation on the landfill site that reflected more Gram-positive organisms than Gram-negative. The morphology also showed that various forms of bacteria, such as rods and cocci, were present.

### 3.2 Qualitative and quantitative screening of *Bacillus* strains for cellulolytic activity using the CMC assay

**Figure 1:**
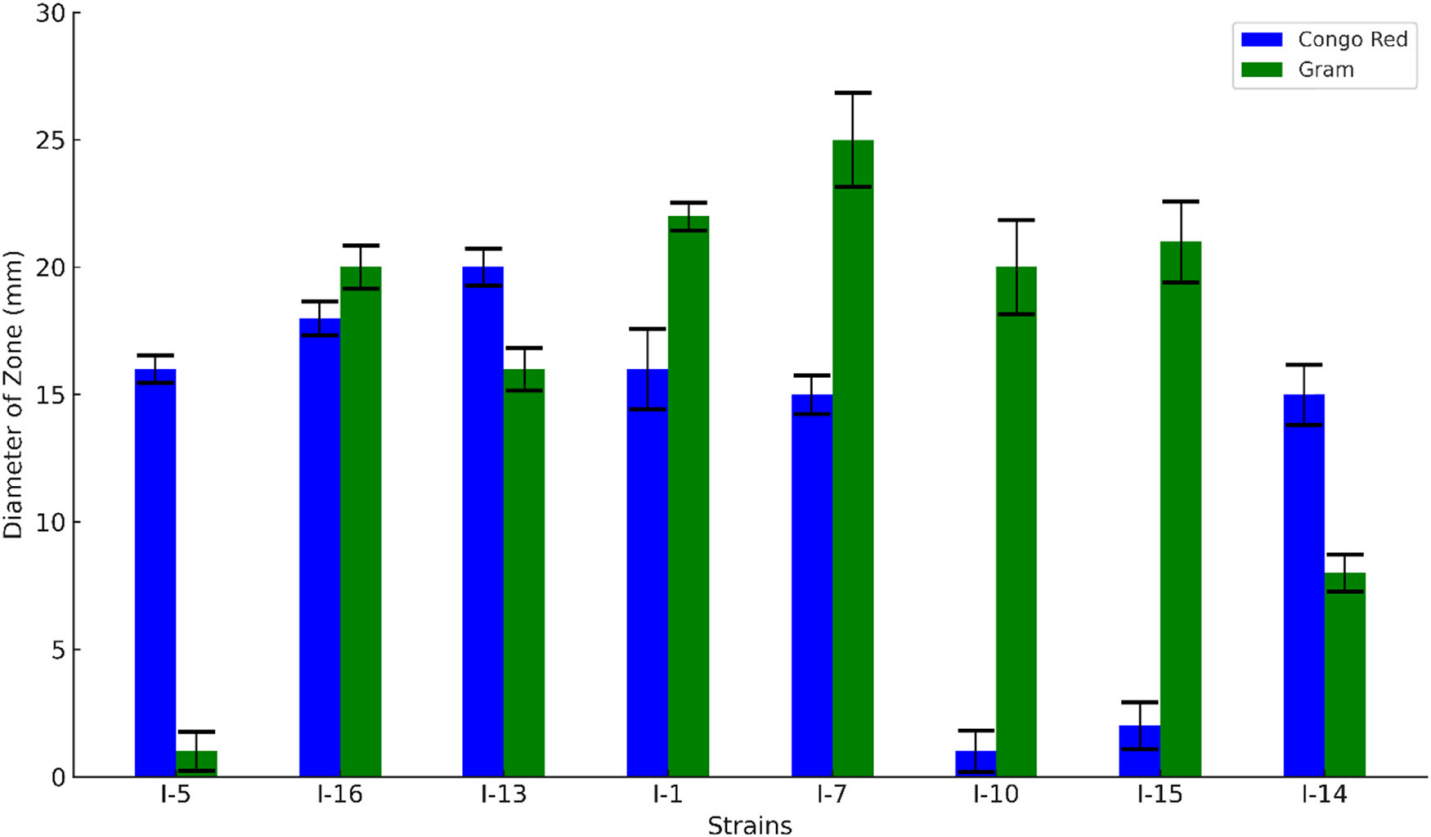
Zones of clearance of different bacterial strains with Congo red and Gram’s iodine test.

The Congo red and Gram’s iodine tests were applied to study the cellulolytic ability of each bacterial strain. Qualitatively, only eight isolates showed significant cellulolytic ability when exposed to Congo red and Gram’s iodine reagent. The other 11 bacterial strains showed hydrolysis and cellulolytic capacity either for only one reagent or not at all. This led to a focus on the eight strains that had positive reactions in the tests. The quantitative reaction of these strains was then studied, and the results for the eight strains are presented in Figure 1.

### 3.3 Effect of temperature on cellulase activity

**Figure 2:**
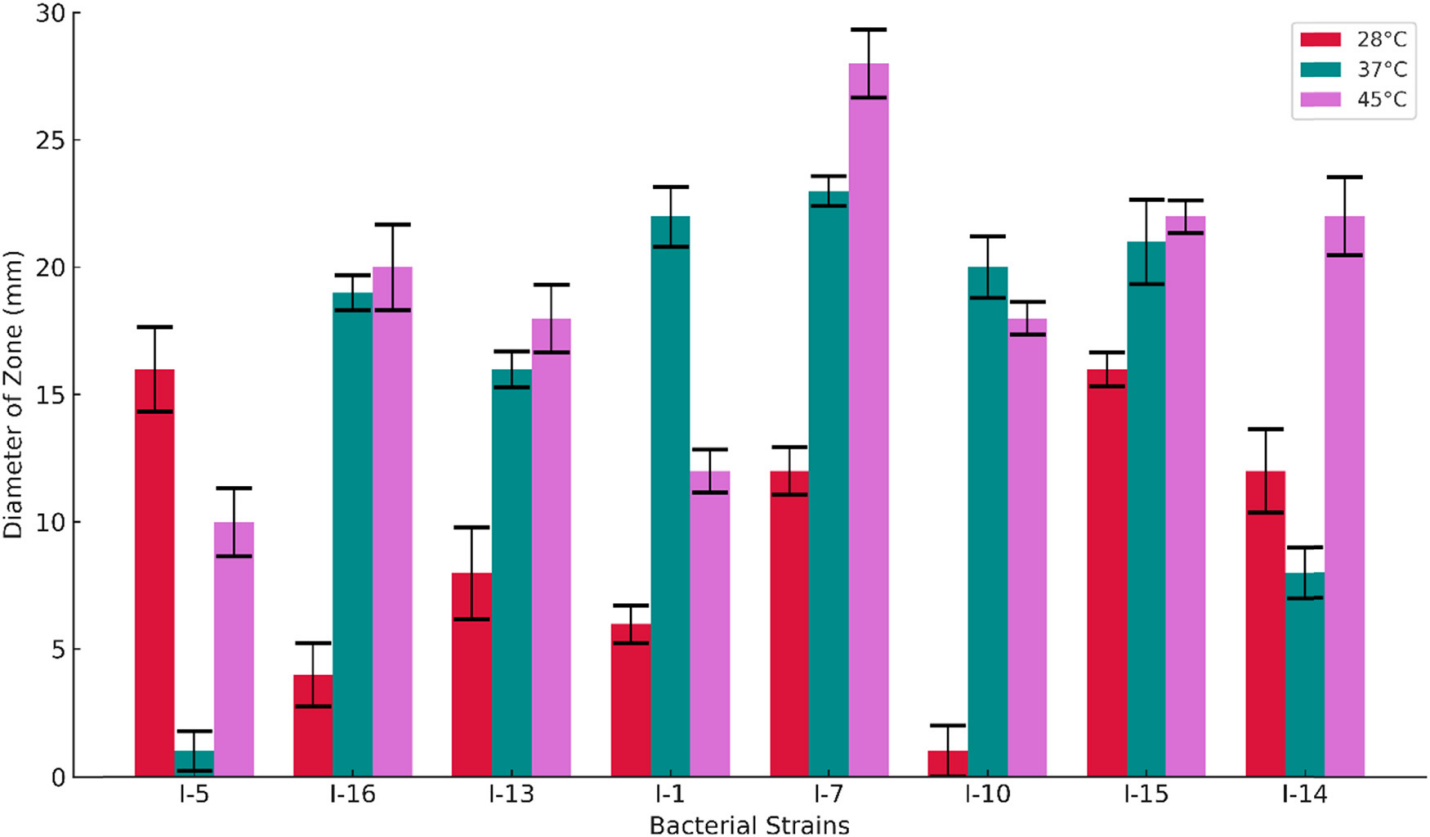
Effect of temperature on the cellulolytic activity of bacterial strains.

The effect of temperature on the qualitative response of the strains was studied and is shown in Figure 2. The results showed that bacterial strains thrive mostly at temperatures above 28°C. Bacterial strains such as I-16, I-13, I-1, I-7, I-10, I-15, and I-14 had more zones of clearance after growth at higher temperatures. However, I-5 was the only strain with a better zone of clearance at 28°C.

### 3.4 Molecular characterization and phylogenetic analysis

The results derived from 16S rDNA sequencing formed the basis of the phylogenetic analysis, which enabled the identification of bacteria that were promising in enzymatic capabilities. In the present study, *Bacillus* genera were identified as a prominent bacterial strain. Table 1 presents the phylogenetic results.

**Table 1:** Culture and molecular-based identification of *Bacillus* strains using 16S rRNA gene sequences obtained from Pulau Burung landfill leachate samples

Sample ID	Coverage	Similarity	BP	Accession	Matched bacteria from NCBI
I1	99	97.33	1203	OQ290603	*B. cereus*
I16	95	99.41	1234	OQ288267	*B. pumilus*
I13	98	99.25	1216	OQ290884	*B. altitudinis*
I5	97	99.74	1168	OQ290885	*B. subtilis*
I7	98	98.62	1226	OQ290886	*B. paramycoides*
I10	93	96.71	1278	OQ290887	*B. niacini*
I15	94	98.75	1215	OQ290888	*B. thuringiensis*
I14	95	97.81	1207	OQ290889	*B. paramycoides*

### 3.5 Growth curve

**Figure 3:**
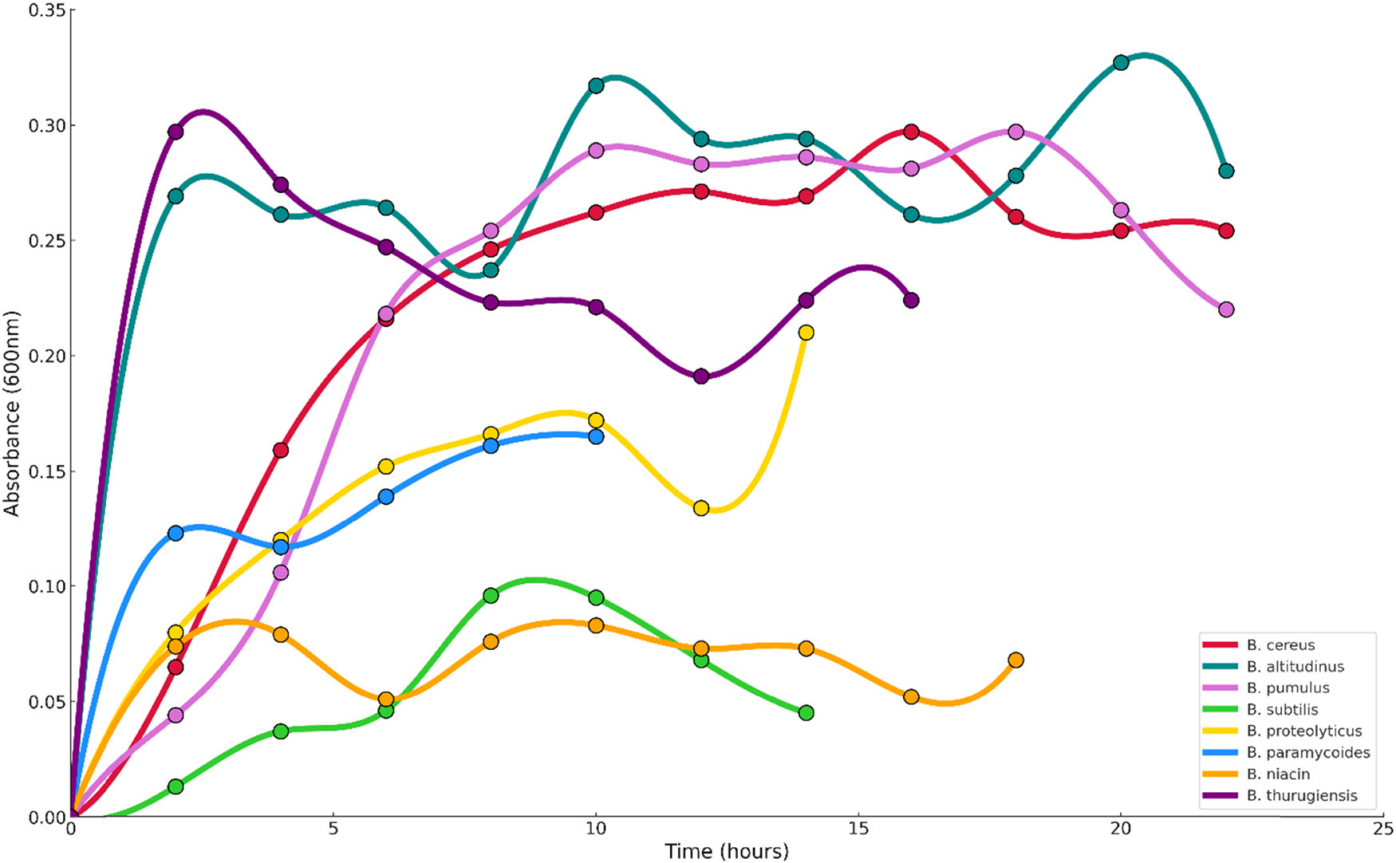
Growth curves of the bacterial strains at 37°C.

**Figure 4:**
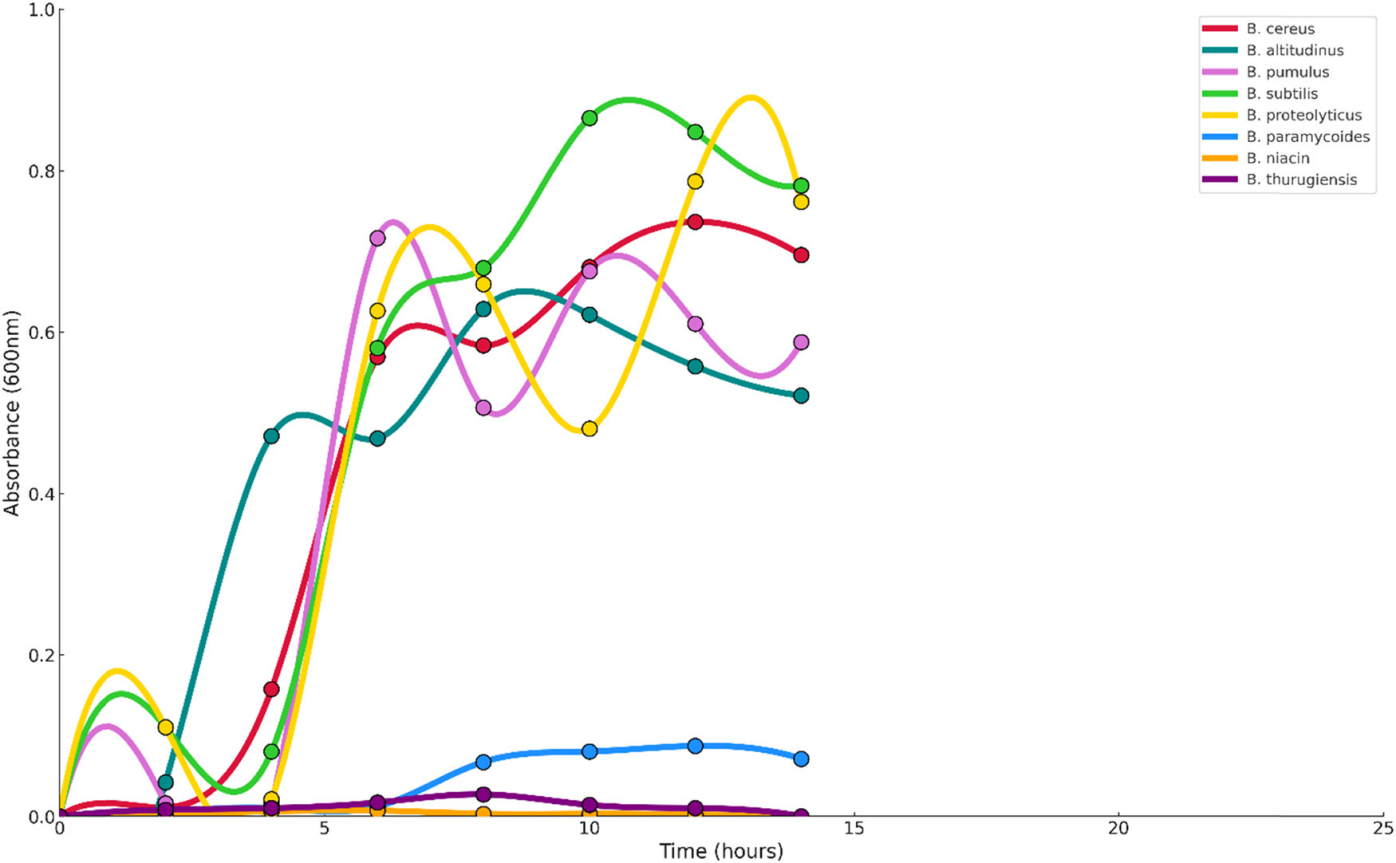
Growth curves of the bacterial strains at 45°C.

For assessment of growth patterns of *Bacillus* strains, they were inoculated in nutrient broth and studied at different temperatures. The flasks were agitated at 150 rpm, and the absorbance was measured at 600 nm for 24 h using a UV-Vis spectrophotometer. The results are shown in Figures 3 and 4.

### 3.6 Quantitative screening and optimization of cellulolytic abilities of the bacterial strains and consortia

**Figure 5:**
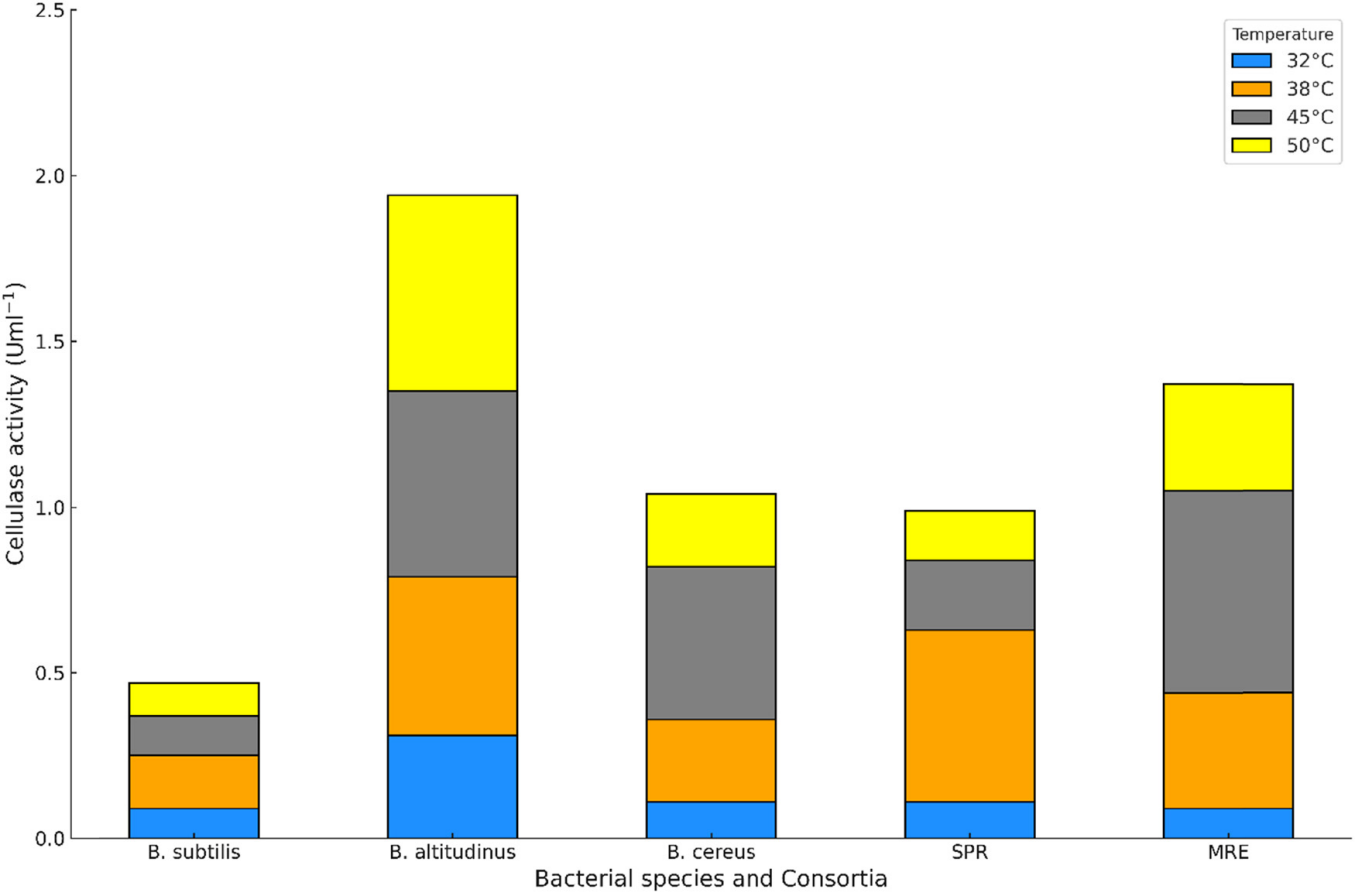
Cellulase activity of the selected bacterial strains and the two consortia at pH 6.

**Figure 6:**
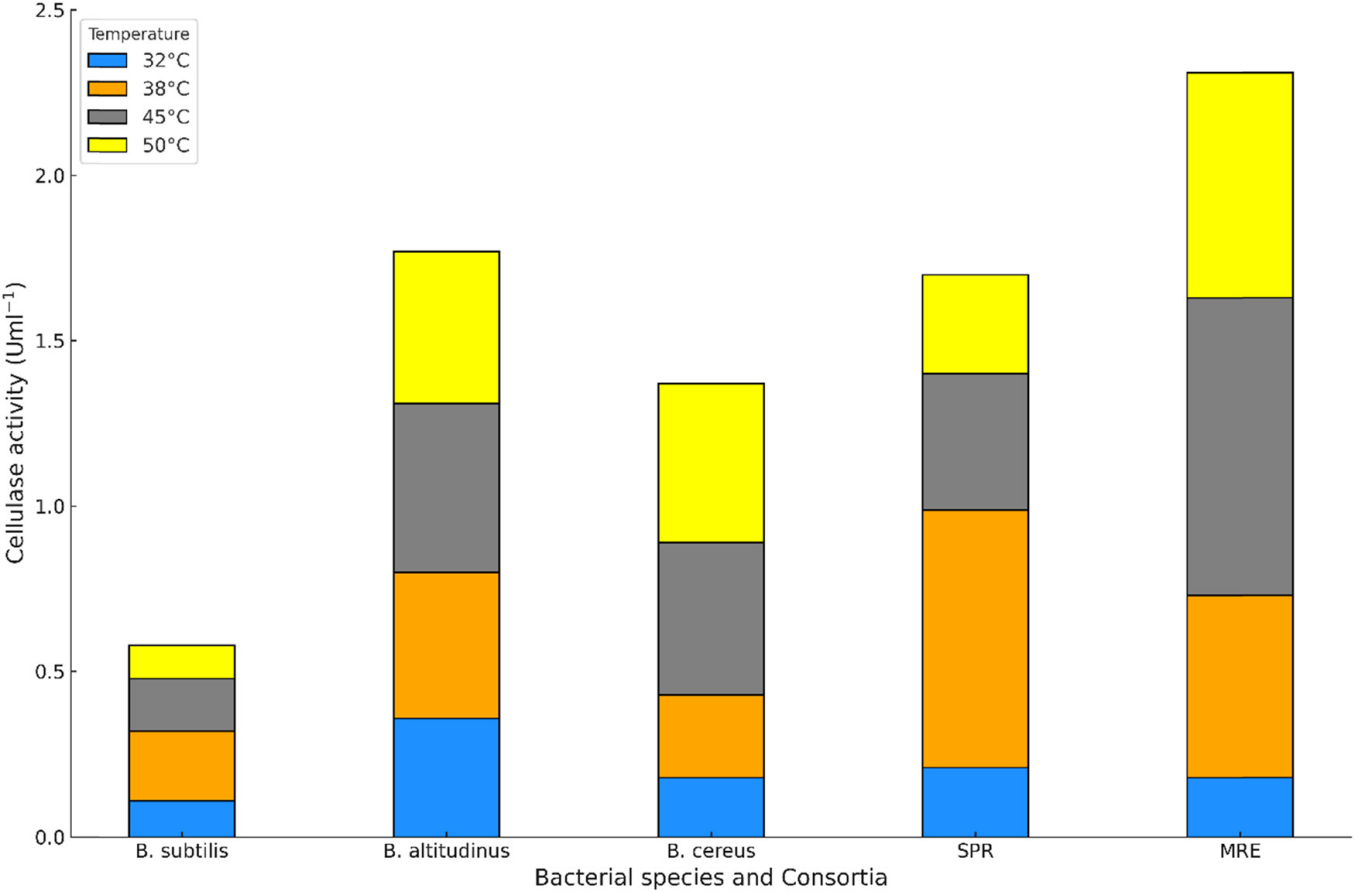
Cellulase activity of the selected bacterial strains and the two consortia at pH 8.

Cellulase activity assays were carried out and optimization was done to check the impact of pH and temperature on cellulase activity. The results are presented in Figures 5 and 6. The assay was also used to check for the cellulolytic improvements in the two consortia that were constructed. The consortia results showed that pH 6 and a temperature of 45°C had the highest results for consortia A, while consortia B had the highest activity at 38°C and pH 6. *B. subtilis* at 32°C had a low response at both pH 6 and 8. While *B. altitudinis* reflected a better response at both pH values used, it exhibited a more significant response at pH 8 than consortia B. At pH 8, consortia A had better activity at higher temperatures of 45 and 50°C, while consortia B showed higher activity at lower temperatures. Both consortia had very similar responses at 32°C. All strains and consortia showed better activity at pH 6, which is an indication that the same pH can be used for further studies.

### 3.7 Optimization of the biodegradation process using RSM

**Figure 7:**
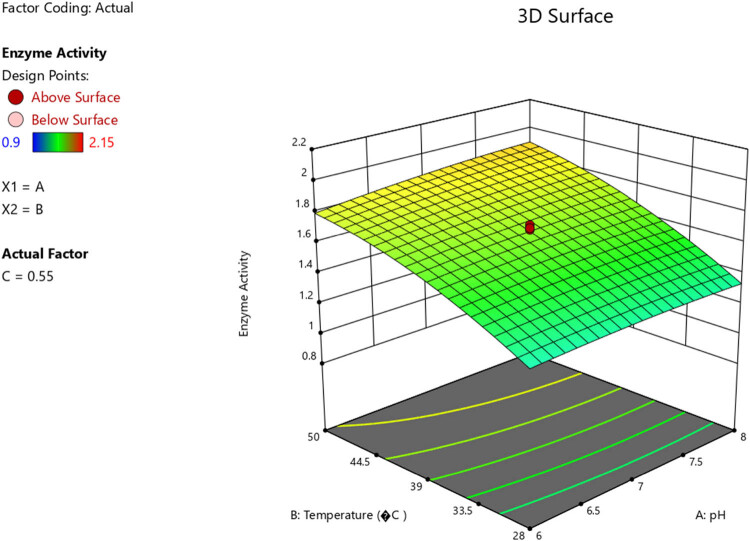
Three-dimensional (3D) response surface diagram reflecting a significant impact of temperature and pH on the enzyme activity.

**Figure 8:**
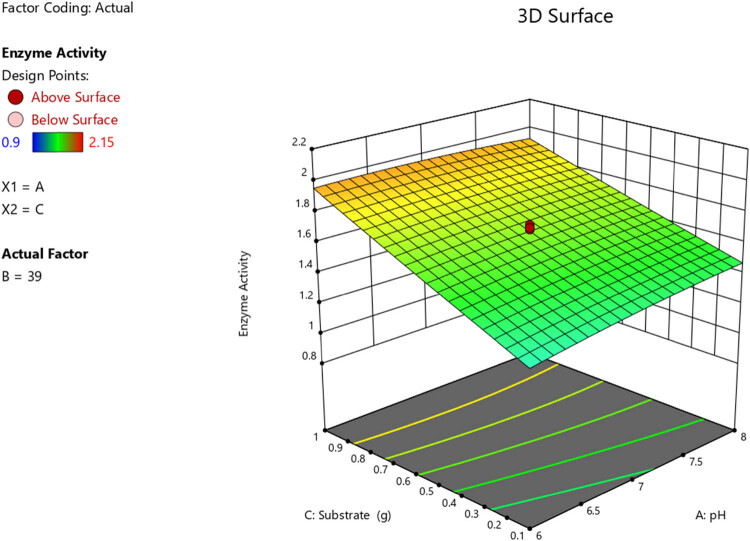
3D response surface diagram reflecting the impact of substrate and pH on the enzyme activity.

A response surface diagram was applied for optimization of the enzyme activity. RSM is a statistical parameter, which analyses different variables concomitantly. This multivariate approach showed benefits like a reduction in investigational trials, increased statistical justification potential, and specified interaction of parameters. A central composite design was applied to examine the optimum state for maximum cellulase generation from consortia. Twenty experimental runs were conducted to analyse the impact of temperature, pH, and substrate on cellulase activity. ANOVA and regression analysis were used in testing for statistical significance. The results showed an insignificant lack of fit test (*F*-values), which shows that the model can satisfactorily predict a response. An *R* ^2^ value of 0.9724 further justified the models. The predicted *R* ^2^ of 0.9167 agreed with the estimated *R* ^2^ of 0.9724, which depicts the adequacy of the model to predict the response. Models were applied to analyse the impact of the independent variables on the response parameters, and the results are presented in Figures 7 and 8. *P*-values that were <0.0500 showed that the model terms were significant.

## 4 Discussion

In this study, after isolation and gram testing, a greater degree of isolates were found to be Gram-positive. The same observations were observed by Garcete et al. [39], as they also found Gram-positive isolates to be more rampant in their investigations. The qualitative screening using Congo red and Gram’s iodine tests revealed eight isolates with significant cellulolytic ability. The zone of clearance reflected cellulase activity, and the diameter of the zone served as an indication of the hydrolysis of cellulose. These eight bacterial strains showed hydrolysis and cellulolytic capacity for both Congo red and Gram’s iodine reagents. After these results were obtained, the focus was narrowed to only those bacterial strains that showed detection zones with both Congo red and Gram’s iodine test. Similar findings were reported by Vu et al. [30], where the bacterial strains showed activity similar to that of Congo red. Approximately 40% of the bacterial isolates showed cellulolytic activity, which was the same as that carried out by Shamshitov et al. [40], who characterized bacteria in a soil environment. Cellulases are enzymes that drive the hydrolysis of cellulosic biomass. Endoglucanase, which is the enzyme activity captured above, is vital in how the hydrolytic process starts and is controlled throughout the process. Thus, its characterization and quantification are imperative to successfully degrade cellulose and apply the results in the industry [41].

Temperature plays a pivotal role not only in bacterial growth but also affects their physiology and enzyme production. Temperature affects the growth and extracellular enzyme generation as it modifies the physical characteristics of the cell membrane. The optimal temperature for growth and cellulase activity is dependent on the microbial strain and isolation site [42]. In the present study, seven out of the eight bacterial strains showed better activity at higher temperatures. Dobrzyński et al. [5] reported similar results, where bacterial strains showed more expression of cellulolytic activity at high temperatures. Islam et al. [43] reported that the *Bacillus* strain showed maximum cellulase production at 35°C, but the enzyme production decreased when the fermentation temperature was increased to above 40°C. This makes the results attractive for industrial purposes due to the possibility of application of thermophilic cellulolytic enzymes in textile, biofuel, and agricultural industries.

Molecular characterization and phylogenetic analysis revealed that the strains that showed promise were from the *Bacillus* genera. *Bacillus* has been reported as a promising bacterial strain with the potential to survive under environmental stresses with its simple nutritional requirements for growth and replication. Their thermophilic nature has also been observed, along with their hydrolytic enzymes like protease and α-amylase generation capacity [44]. The potential bacterial strains identified were *B. proteolyticus, B. pumilus, B. paramycoides, B. cereus, B. subtilis, B. niacini, B. altitudinis,* and *B. thuringiensis.* The assessment of the growth pattern of the *Bacillus* strains was done at different temperatures. The growth curve profile showed increased cell activity at 45°C from hour 5 for most of the bacterial strains, except for *B. paramycoides*, *B. niacini*, and *B. thuringiensis*. The growth studies confirmed that these *Bacillus* strains can utilize cellulose as a main source of carbon for their growth and replication. Bacteria tend to grow best at optimal temperatures of 30–37°C [45].

The DNS assay results from this study reflected that the *Bacillus* strains showed tangible cellulolytic potential, which agrees with the study conducted by Pandey et al. [46], where the *Bacillus* species showed potential in enzyme production by using several cellulosic substrates. The isolates were also able to remain active under different pH and temperature ranges. This attribute implies that bacterial species can be used for the production of thermostable enzymes, which was cost-effective due to the simultaneous saccharification and fermentation of lignocellulosic wastes into biofuel and other value-added products. Balla et al. [47] reported that the catalase enzyme activity was 0.25 U/ml at pH 6 by the *B. pumilus* EWBCM1 strain.

The co-cultures are advantageous, as microbes that are in proximity reflect significant interactions, which are beneficial for them. Earlier reports revealed that tasks that single cultures might find tedious due to more consumption time were easily achieved by consortia related to bioremediation and generation of bioproducts [30]. This makes microbial consortia a vital part of the design process of models that comprise up to four species, which can be used to sufficiently predict environmental interactions and behaviour between microorganisms [48]. This assisted in the design of consortia used for further investigations. The high temperature favoured cellulolytic activity in the consortia, and *Bacillus* strains with pH 6 indicated higher cellulolytic activity for most of the bacterial isolates and consortia. These results are similar to the findings observed by Dar et al. [49], as they showed that pH and temperature affected the metabolism and catalytic efficiency of *Bacillus* species. Ilic et al. [50] reported that the pH of the medium plays an important role in cellulase generation.

Their results showed that maximum cellulase production was observed at pH 5 and 50°C. It is similar to our results, where pH 6 and 45°C had the highest results for consortia A, and consortia B had the highest activity at 38°C. In our results, pH 6 was very more favourable for cellulolytic activity compared to pH 8 for both the strains and consortia. The affinity for high temperatures and pH 6 is similar to the study on *B. coagulans* by Aulitto et al. [51], where bacteria secreted cellulases at 37°C; however, the optimal temperature was 50°C and pH was 6. Consortia A showed more consistent results with varying pH and temperature and was selected for further study. Consortia are also advantageous, as existing limitations of single strains, like the metabolic burden of gene overexpression, toxin, inhibition by intermediates, and thermodynamic challenges, can be overcome using the principle of division of labour. Co-culturing can help in reducing steps involved during pre-treatment and enzyme production and can be integrated into one step to reduce expenditure [52].

RSM was used for the optimization of the enzyme activity for consortia A, which showed the most significant results at high temperatures. After 20 experimental runs were done to show the impact of temperature, pH, and substrate on the activity of cellulase, statistical analysis showed that the model was adequate for predicting the response being studied. ANOVA and regression analysis were used in testing for statistical significance. Model terms B, C, AC, and B² were significant, whereas values >0.1000 showed that model terms were not significant. The results were confirmed, which reflected that the consortia’s optimum conditions were pH 7 and a temperature of 39°C.

## 5 Conclusions

In this investigation, we have isolated and identified bacteria with high enzymatic ability from landfill leachate of the Pulau Burung landfill site of Penang, Malaysia. The eight most significant isolates obtained were *Bacillus* species, which showed cellulolytic ability with both Congo red and Gram’s iodine test. Bacterial isolates were utilized in the formation of two consortia, A and B, which were screened, and the results showed that both consortia performed better s compared to individual strains, and consortia A showed better potential under varying conditions. Hence, co-cultures are beneficial as they can synergistically promote steps of the bio-conversion process and may result in a tangible reduction of cost leading to more adoption of biological procedures for biorefinery development. The biodegradation of lignocellulose biomass by bacterial enzymes is a promising and sustainable approach, as bacterial cellulase can simultaneously perform the role of pre-treatment and easily break down recalcitrant lignocellulosic components. Cellulolytic enzymes are widely used in the paper, pulp, and textile industries. However, the use of cellulase in biofuel production is expected to increase and replace around 30% of fossil fuels by the year 2025. Introducing specific mutations from one hyper-cellulolytic strain into another is a feasible strategy for improving cellulase production for industrial applications. Further investigations are required to explore the potential bacterial species or consortia or the development of engineered *Bacillus* strain, which may assist in the low-cost generation of cellulase for various commercial applications.
